# Comparing the Microbial Community in Four Stomach of Dairy Cattle, Yellow Cattle and Three Yak Herds in Qinghai-Tibetan Plateau

**DOI:** 10.3389/fmicb.2019.01547

**Published:** 2019-07-10

**Authors:** Jinwei Xin, Zhixin Chai, Chengfu Zhang, Qiang Zhang, Yong Zhu, Hanwen Cao, Jincheng Zhong, Qiumei Ji

**Affiliations:** ^1^State Key Laboratory of Hulless Barley and Yak Germplasm Resources and Genetic Improvement, Lhasa, China; ^2^Institute of Animal Science and Veterinary, Tibet Academy of Agricultural and Animal Husbandry Sciences, Lhasa, China; ^3^Key Laboratory of Qinghai-Tibetan Plateau Animal Genetic Resource Reservation and Utilization, Sichuan Province and Ministry of Education, Southwest Minzu University, Chengdu, China

**Keywords:** Qinghai-Tibetan Plateau, yak, foregut, host genetics, geography

## Abstract

Yak (*Bos grunniens*) is an unique ruminant species in the Qinghai-Tibetan Plateau (QTP). The ruminant gastrointestinal tract (GIT) microbiota is not only associated with the nutrients metabolism, but also contributes to the host’s local adaptation. Examining the microbiota between cattle and yak in different geography could improve our understanding about the role of microbiota in metabolism and adaptation. To this end, we compared the microbiota in rumen, reticulum, omasum, and abomasum of dairy cattle, yellow cattle, and three yak herds (WQ yak, SZ yak, and ZB yak) lived in different altitude, based on sequencing the bacterial 16S rRNA gene on Illumina Miseq. The bacterial diversity was significantly different among five breeds, whereas the difference among four stomach regions is limited. The phyla *Bacteroidetes* and *Firmicutes* were the dominated bacteria regardless of breeds and regions. The nonmetric multidimensional scaling (NMDS) results showed that the microbiota in dairy cattle, yellow cattle and WQ yak significantly differed from that in SZ yak and ZB yak for all four stomach compartments. Canonical correlation analysis revealed that *Prevotella* and *Succiniclasticum* spp. were abundant in dairy cattle, yellow cattle and WQ yak, whereas the *Christensenellaceae* R7 group and the *Lachnospiraceae* UCG 008 group were prevalent in SZ yak and ZB yak. Moreover, the microbiota in WQ yak was significantly different from that in SZ yak and ZB yak, which were characterized by the higher relative abundance *Romboutsia* spp., *Eubacterium coprostanoligenes*, *Acetobacter* spp., *Mycoplasma* spp., and *Rikenellaceae* RC9 group. Overall, these results improves our knowledge about the GIT microbiota composition of QTP ruminant.

## Introduction

The Ruminantia taxon is one of the most important groups of large terrestrial herbivorous mammals and is distributed over a wide geographical range, from the Qinghai-Tibetan Plateau (QTP) to the North Pole, and includes animals such as reindeer and muskox (Fernández and Vrba, 2005). Additionally, the domesticated ruminants are also particularly important in the agricultural system because they provide meat and milk for human consumption (Eisler et al., 2014). Of the domesticated ruminants, Yak (*Bos grunniens*) is a unique and remarkable species in the QTP region adapted to the low temperature and oxygen level (3,000–5,500 m) and has lived with people more than 7,000 years (Qiu et al., 2015). QTP yaks also play an essential and beneficial role in local human civilization and agricultural development as they provide basic resources for survival, including transportation, dung for fuel and hides for tents (Wiener et al., 2003; Qiu et al., 2012). Although in nature yak graze the natural grassland throughout the year with limited supplementation of concentrated feed, they are seriously challenged by poor foraging resources in the QTP region during the senescent season (Long et al., 2005). Therefore, yak must have evolved the underlying mechanisms to adapt to the harsh environment.

Genome analysis has improved our understanding of the adaptation of yak (Qiu et al., 2012); however, recent studies have documented that the trillions of microbial cells living in the gastrointestinal tract (GIT), termed microbiota, also play vital roles in host adaptation, showing an eco-evolutionary relationship with the host (Brooks et al., 2016; Moeller et al., 2016; Kwong et al., 2017; Ross et al., 2018; Zepeda Mendoza et al., 2018). For ruminants, there are enlarged foregut regions, including four compartments named the rumen, reticulum, omasum, and abomasum, with different functions (Aschenbach et al., 2011). The ingested feed is mainly fermented in the reticulo-rumen, which provides suitable anaerobic conditions for microorganisms (Aschenbach et al., 2011). Salts and water are absorbed in the omasum, whereas the undigested feed is further broken down by enzymes and acids in the abomasum. Among the four compartments, the diverse and complex microbiota in the rumen, particularly bacteria, play a fundamental role in nutrient metabolism (Wu et al., 2016) by converting protein and plant fibers into volatile fatty acids and microbial proteins and supplying essential nutrients and energy to the host. In previous studies, rumen bacteria (An et al., 2005; Dai et al., 2012; Guo et al., 2015; Zhang et al., 2016; Zhou et al., 2017) and methanogens (Huang et al., 2012) have been extensively examined; however, knowledge regarding the microbiota in the four foregut compartments of yak is very limited (Xue et al., 2018). Additionally, the GIT microbiota is significantly affected by host genetics (Goodrich et al., 2014) and geographic location (Moeller et al., 2013). Therefore, it is hypothesized that the foregut microbiota in the different ruminant species and locations will be distinct.

In this study, we compared the microbiota in the rumen, reticulum, omasum, and abomasum of Holstein cattle (dairy cattle, 616 m), Sanjiang cattle (yellow cattle, 1,484 m), Leiwuqi yak (WQ yak, 4,500 m), Shenza yak (SZ yak, 4,700 m) and Zhongba yak (ZB yak, 4,700 m) based on the sequencing of the bacterial 16S rRNA gene with the Illumina Miseq platform. Our results demonstrated that the genera *Prevotella* and *Rikenellaceae* RC9 were the abundant bacteria in the four stomach chambers of cattle and yak. The stomach microbiota of SZ yak and ZB yak are significantly different from that of dairy cattle, yellow cattle and WQ yak. Moreover, the microbiota of WQ yak also differed from that of SZ yak and ZB yak. These results suggested that host genetics and geography affected the stomach compartment microbiota. These results not only enhance our knowledge about the role of microbiota in the nutrition and metabolism of yak, which thus provides new insights into nutrition management, but also improve our understanding of yak adaptation.

## Materials and Methods

### Animals and Sample Collection

In this study, three female Holstein cattle (dairy cattle) in lactation from Taiping Village, Lichun Town, Pengzhou City, Sichuan Province (BW = 674.0 ± 25.2, Age = 4.5 years old), three female Sanjiang cattle (yellow cattle) in Maliu Village, Sanjiang Town, Wenchuan County, Chengdu City, Sichuan Province, China (BW = 197.7 ± 15.6, Age = 4.5 years old), three female WQ yak in Riwoqe County, Qamdo City, Tibet Autonomous Region (BW = 210.4 ± 33.5, Age = 4.5 years old), three female SZ yak in Shenza County, Nagchu City, Tibet Autonomous Region (BW = 156.0 ± 26.7, Age = 4.5 years old), and three female ZB yak in Zhongba County, Shigatse City, Tibet Autonomous Region (BW = 200.8 ± 17.1, Age = 4.5 years old) that were raised by local farmers were used (Supplementary Table S1). The animals were purchased from the local herdsman. The Sanjiang cattle and three yak herds were pastured on grassland without supplementary feed and housing from September to November, 2017. The animals were slaughtered according to the strict procedures and guidelines of the Institute of Animal and Veterinary Science, Tibet Academy of Agricultural and Animal Husbandry Sciences. In order to minimize the potential contamination among the four compartments, we placed the carcass in a natural position. After that, each stomach chambers were tied off using cotton rope, and were transferred to the sterilized brown paper. Then, the contents from different chambers of the rumen, reticulum, omasum, and abomasum from each animal were carefully collected. The contents were homogenized using sterile gloves, and then approximately 200 g contents were stored in the DNases and RNases free tubes. A total of 60 samples were collected, immediately frozen in liquid nitrogen and stored at −80°C for further analysis.

All animal-specific procedures were approved and authorized by the Tibet Academy of Agricultural and Animal Husbandry Sciences Animal Care and Use Committee.

### DNA Extraction, PCR Amplification, and High-Throughput Sequencing

The microbial genomic DNA was extracted from each sample using the QIAamp DNA Stool Mini Kit (QIAGEN, Valencia, CA, United States) according to the manufacturer’s instructions. The primers 341F (5′-CCTAYGGGRBGCASCAG-3′) and 806R (5′-GGACTACHVGGGTWTCTAAT-3′) were used to amplify the bacterial 16S rRNA gene V3-V4 region (Human Microbiome Project Consortium, 2012). The resulting amplicons were then sequenced on an Illumina PE MiSeq 250 platform. The pair ended sequences for each sample were assembled into contiguous sequences (contigs) using FLASH (Magoč and Salzberg, 2011). The contigs were then treated for quality control using the following criteria: the minimum quality score was 25; the maximum number of errors in the barcode was 0; the allowed maximum length of homopolymer was 6; the number of mismatches in the primer was 0. The sequences with any ambiguous and unassigned characters were also removed. The generated sequences were then analyzed using QIIME 1.9.0 (Caporaso et al., 2010). In brief, the sequences were clustered into operational taxonomic units (OTUs) using UPARSE at 97% sequence identity (Edgar, 2013) after quality control. UCHIME was applied to remove potential chimeric sequences using the *de novo* parameter (Edgar et al., 2011). Representative sequences of the OTUs were used for taxonomic classification using the SILVA database (version 123) (Wang et al., 2007; Quast et al., 2013). Finally, we rarefied the data of each sample to the minimum numbers (8,497) after removing the singletons. The alpha-diversity indices, including Shannon and Chao1, were calculated using QIIME 1.9.0 (Caporaso et al., 2010).

### Bioinformatics and Statistical Analyses

Nonmetric multidimensional scaling analysis based on unweighted UniFrac distance and Bray–Curtis distance was applied to compare the microbiota in different regions across five breeds. To test the similarities among the microbiota across all samples, analysis of similarities (ANOSIM) using 999 permutations was performed based on the Bray–Curtis distance in the R environment. The ANOSIM test statistic R indicates group similarity, where 0 = indistinguishable and 1 = dissimilar. Canonical correlation analysis (CCA) was then also applied to identify the representative bacteria at the genus level of each sample based on the indicator species analysis using the RAM package (Dufrene and Legendre, 1997). The indicator species analysis selected the most representative features for each cluster or group and split these features into the number of clusters being compared. Associated taxa were identified by assigning an indicator value to each taxon. This indicator value was the product of the relative average abundance and relative frequency of that feature in a group. The indicator values closer to one (1.0) suggest a high abundance of a feature within a group as compared to others. Moreover, the significance of the indicator value is evaluated by permutation tests (probability). In this study, we used a indicator value >0.5 for discriminant taxa. All comparisons of the alpha-diversity indices and the bacterial abundance were analyzed using a one-way ANOVA, and Tukey’s multiple comparisons test was used in multiple comparisons using of SPSS22 (SPSS, Chicago, IL, United States). All *p*-values were corrected for a false discovery rate (FDR) of 0.05 using Benjamini–Hochberg. FDR-corrected *p-*values below 0.05 (FDR < 0.05) were considered significant. All values were expressed as the mean unless otherwise stated.

## Results

### Summary of Sequencing and Comparison of the Diversity Indices in the Foregut of Five Breeds

In the present study, we obtained a total of 3,165,932 high quality 16S rRNA gene sequences in the range of 8,106–85,936 sequences per sample. To decrease the influence of sequencing depth on the microbiota identification, we sub-sampled the data from each sample to the minimum member (8,497).

A total of 27,342 OTUs were identified in the four stomach chambers based on 97% sequence identity, ranging from 1,894 to 6,128 OTUs per sample. Good’s coverage was greater than 57% (Table 1), suggesting that much more microbial species was presented in the stomach. Comparing the diversity indices among the five breeds showed that the Shannon diversity index in the four stomach chambers of dairy cattle, yellow cattle and WQ yak was higher than that in SZ yak and ZB yak (Table 1).

**TABLE 1:** Comparison of the alpha diversity indices in the different foregut regions across five breeds.

**Host**	**Rumen**				**Reticulum**				**Omasum**				**Abomasum**			
	**OTUs**	**Coverage**	**Shannon**	**Chao1**	**OTUs**	**Coverage**	**Shannon**	**Chao1**	**OTUs**	**Coverage**	**Shannon**	**Chao1**	**OTUs**	**Coverage**	**Shannon**	**Chao1**
Dairy	3,640	0.72^*ab*^	7.5^*ab*^	8,894	3,699	0.71	7.5	9,226	3,698	0.71	7.4	9,231	3,379	0.74	7.2	8,285
Yellow	4,778^a^	0.64^a^	7.8^a^	16,255^a^	5,327^a^	0.57^b^	8.0^a^	18,801	3,244	0.74	7.1	8,383	4,021	0.65	7.4	14,789
WQ	3,699	0.71^*ac*^	7.4^*ac*^	8,900	3,337	0.74	7.2	8,458	3,315	0.73	7.1	8,441	3,202	0.75	7.1	7,943
SZ	3,215^b^	0.74	7.0	8,940	3,976	0.65	7.4	16,053	3,219	0.73	7.0	8,714	2,880	0.77	6.6	7,790
ZB	2,693^b^	0.78^b^	6.4^b^	7,241^b^	2,791^b^	0.78^a^	6.6^b^	7,378	2,782	0.76	6.6	7,444	2,580	0.79	6.1	7,128
SEM	208	0.02	0.2	1,042	272	0.03	0.2	1,546	115	0.01	0.1	327	176	0.01	0.2	998
*P*	0.002	0.003	0.005	0.02	0.01	0.02	0.008	0.03	0.14	0.30	0.12	0.59	0.07	0.06	0.13	0.07

*Different letters in the same column indicate the significant differences from each other.*

### Bacterial Composition in the Rumen, Reticulum, Omasum, and Abomasum of Dairy Cattle, Yellow Cattle, and Three Yak Herds

**FIGURE 1:**
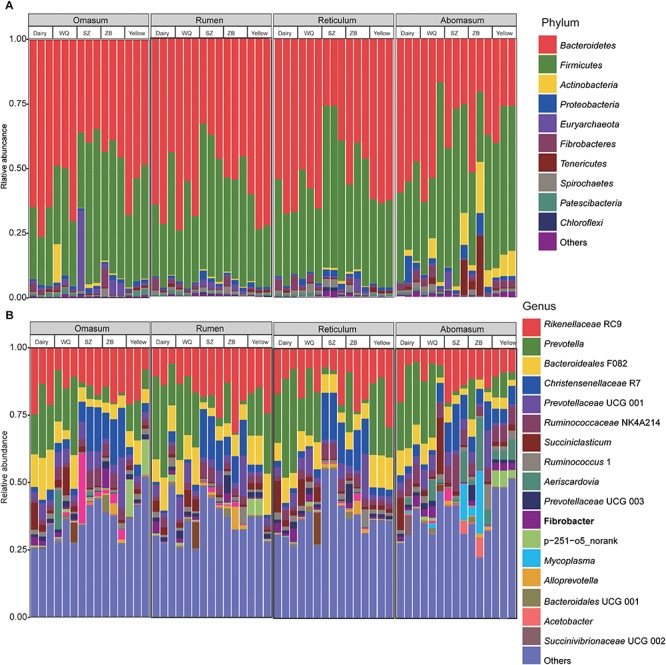
Bacterial composition at the phylum **(A)** and genus levels **(B)** in the rumen, reticulum, omasum, and abomasum of cattle and three yak herds. Dairy = dairy cattle, Yellow = yellow cattle, WQ = WQ yak, SZ = SZ yak, ZB = ZB yak.

In the rumen, a total of 38 phyla were identified from the 15 samples, ranging from 19 to 36 phyla for dairy cattle, yellow cattle, and yaks (Figure 1A). The phyla *Bacteroidetes* (dairy cattle = 60.2%, yellow cattle = 64.8%, WQ yak = 66.3%, SZ yak = 39.1%, and ZB yak = 51.6%) and *Firmicutes* (dairy cattle = 33.3%, yellow cattle = 29.9%, WQ yak = 28.9%, SZ yak = 52.9%, and ZB yak = 41.7%) were the predominant bacteria in the rumen of all breeds, accounting for approximately 93% of the taxonomic groups identified. Moreover, bacteria belonging to the phyla *Fibrobacteres*, *Spirochaetes*, and *Proteobacteria* were present in all samples at the level of 2.5–4.0%. At the genus level (Figure 1B), *Prevotella* was the most dominant bacterium in the rumen of dairy cattle (26.8%), yellow cattle (18.0%) and WQ yak (23.5%), followed by the *Rikenellaceae* RC9 group (dairy cattle = 10.2%, yellow cattle = 19.3%, and WQ yak = 13.5%) and the *Bacteroideales* F082 group (*Bacteroidales*, dairy cattle = 10.1%, yellow cattle = 12.3%, and WQ yak = 11.9%), accounting for approximately 47% of the overall bacterial composition. However, *Rikenellaceae* RC9 was the most abundant bacteria in the rumen of SZ yak (20.6%) and ZB yak (21.5%), followed by the *Christensenellaceae* R7 group (SZ yak = 15.1%, ZB yak = 10.0%).

In the reticulum, a total of 40 phyla were observed in all samples, ranging from 16 to 40 phyla per sample (Figure 1A). The phylum *Bacteroidetes* was the most abundant bacteria in the reticulum of dairy cattle (63.0%), yellow cattle (62.5%), WQ yak (58.1%), and ZB yak (47.7%), followed by the phylum *Firmicutes* (dairy cattle = 29.9%, yellow cattle = 32.5%, WQ yak = 34.4%, and ZB yak = 44.0%), which accounted for more than 92% of all bacterial taxa. However, the dominant bacteria in the reticulum of SZ yak was the phylum *Firmicutes* (59.9%), followed by the phylum *Bacteroidetes* (30.3%). At the genus level (Figure 1B), *Prevotella* was also the most dominant bacterium in the reticulum of dairy cattle (31.1%), yellow cattle (21.5%) and WQ yak (18.6%), followed by *Rikenellaceae* RC9 (dairy cattle = 11.8%, yellow cattle = 18.2%, and WQ yak = 13.9%), *Bacteroideales* F082 (dairy cattle = 7.2%, yellow cattle = 12.0%, and WQ yak = 7.1%), and *Succiniclasticum* spp. (dairy cattle = 5.3%, yellow cattle = 1.6%, and WQ yak = 5.6%), which together made up 45% of the bacterial composition. However, *Christensenellaceae* R7 and *Rikenellaceae* RC9 were the most predominant bacteria in the reticulum of SZ yak (16.6%) and ZB yak (17.0%), respectively.

In the omasum, a total of 32 phyla were identified (Figure 1A). The predominant bacteria were in the phylum *Bacteroidetes* (dairy cattle = 68.7%, yellow cattle = 56.7%, WQ yak = 56.3%, respectively), followed by the phylum *Firmicutes* (dairy cattle = 25.6%, yellow cattle = 37.3%, and WQ yak = 32.2%), which accounted for more than 88% of all bacterial taxa. For SZ yak and ZB yak, the phylum *Firmicutes* (SZ yak = 47.9% and ZB yak = 46.9%) was the most abundant bacteria, followed by the phylum *Bacteroidetes* (SZ yak = 36.8% and ZB yak = 42.6%). At the genus level, *Rikenellaceae* RC9 was the prevalent bacteria in the omasum throughout all samples (dairy cattle = 19.7%, yellow cattle = 16.3%, and WQ yak = 11.3%, SZ yak = 14.8% and ZB yak = 17.2%, Figure 1B). *Prevotella* was also abundantly present in the omasum of dairy cattle (19.6%), yellow cattle (10.6%) and WQ yak (21.2%), while the occurrence of *Christensenellaceae* R7 was high in the omasum of SZ yak (13.4%) and ZB yak (15.0%).

In the abomasum, a total of 27 phyla were identified in all samples (Figure 1A). The phylum *Bacteroidetes* was the most abundant phylum in the abomasum of dairy cattle (54.1%) and WQ yak (44.7%), followed by the phylum *Firmicutes* (dairy cattle = 33.3% and WQ yak = 42.9%). In contrast, the phylum *Firmicutes* was much more abundant in the abomasum of yellow cattle (51.5%), SZ yak (52.3%) and ZB yak (38.5%) than other phyla, and the phylum *Bacteroidetes* was the second most abundant. At the genus level, *Prevotella* (dairy cattle = 24.0%, yellow cattle = 6.1%, WQ yak = 18.4%) and *Rikenellaceae* RC9 (dairy cattle = 10.2%, yellow cattle = 9.7%, WQ yak = 7.9%) were abundantly present in the abomasum of dairy cattle, yellow cattle, and WQ yak. However, *Rikenellaceae RC9* was the most abundant bacteria in SZ yak (16.4%) and ZB yak (12.3%).

### Comparing the Microbiota in Dairy Cattle, Yellow Cattle, and Three Yak Herds

**FIGURE 2:**
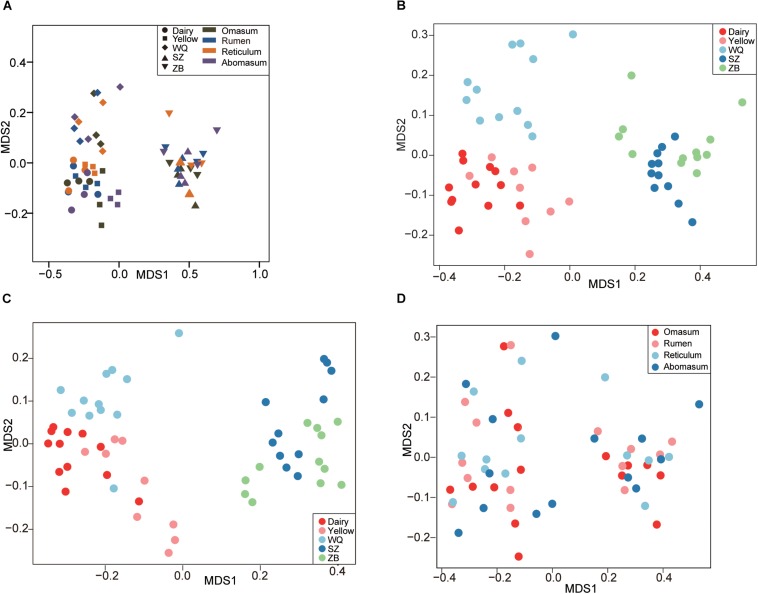
Comparing the stomach microbiota across five breeds. **(A)** Nonmetric multidimensional scaling (NMDS) plot revealing the effects of breeds and stomach regions based on the Bray–Curtis distance. NMDS plots showing the effect of breed on stomach microbiota based on the Bray–Curtis distance **(B)**, and the unweighted UniFrac distance **(C)**. **(D)** Comparing the microbiota among the four stomach chambers based on the Bray–Curtis distance. Dairy = dairy cattle, Yellow = yellow cattle, WQ = WQ yak, SZ = SZ yak, ZB = ZB yak.

**FIGURE 3:**
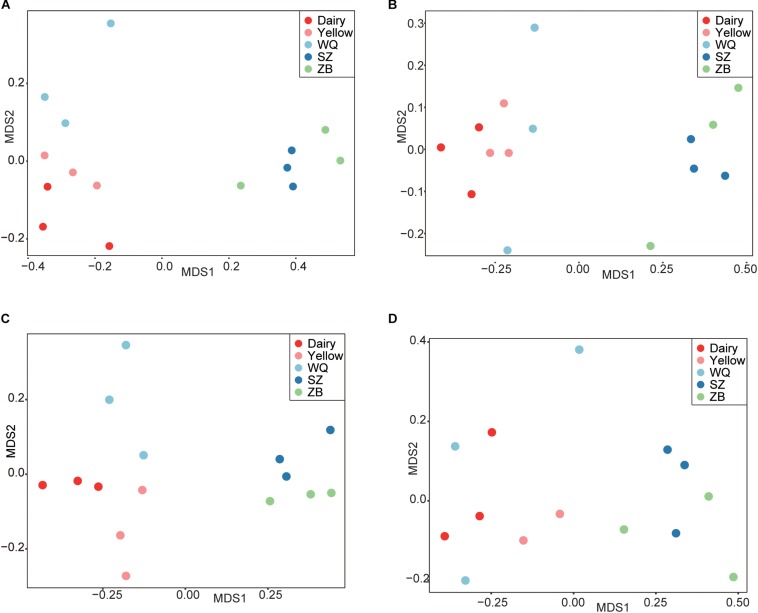
The NMDS plots based on the Bray–Curtis distance to reveal the variation of microbiota in the rumen **(A)**, reticulum **(B)**, omasum **(C)**, and abomasum **(D)** of cattle and three yak herds. Dairy = dairy cattle, Yellow = yellow cattle, WQ = WQ yak, SZ = SZ yak, ZB = ZB yak.

We applied the NMDS analysis of dissimilarities and the ANOSIM test to examine differences in the microbiota composition and structure in the four stomach chambers among the five breeds. We first considered the effects of breeds and stomach regions (Figure 2A), and the results showed that the microbiota was significantly different among all samples based on the Bray–Curtis distance (ANOSIM: *r* = 0.46, *p* = 0.001). Interestingly, the NMDS plot showed that the microbiota separated into two clear clusters across all breeds, one including dairy cattle, yellow cattle, and WQ yak; and another including SZ yak and ZB yak, based on the Bray–Curtis distance (ANOSIM: *r* = 0.61, *p* = 0.01, Figure 2B) and the unweighted UniFrac distance (ANOSIM: *r* = 0.63, *p* = 0.01, Figure 2C). The microbiota was not significantly different between the rumen, reticulum, omasum, and abomasum based on the Bray–Curtis distance (ANOSIM: *r* = 0.01, *p* = 0.55, Figure 2D) and the group distance across all breeds (Supplementary Figure S1). However, the NMDS results based on the Bray–Curtis distance showed that the microbiota in the rumen (ANOSIM: *r* = 0.79, *p* = 0.001, Figure 3A), reticulum (ANOSIM: *r* = 0.78, *p* = 0.001, Figure 3B), omasum (ANOSIM: *r* = 0.83, *p* = 0.001, Figure 3C), and abomasum (ANOSIM: *r* = 0.71, *p* = 0.01, Figure 3D) were significantly separated according to the ruminant breeds.

### Comparing the Microbiota in the Rumen, Reticulum, Omasum, and Abomasum of the Five Breeds

**FIGURE 4:**
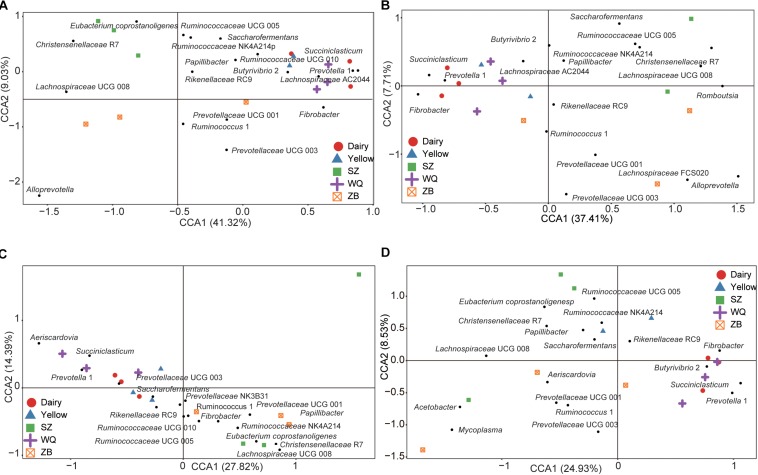
Canonical correlation analysis (CCA) showing the representative bacteria at the genus level in the rumen **(A)**, reticulum **(B)**, omasum **(C)**, and abomasum **(D)** across the five breeds. Dairy = dairy cattle, Yellow = yellow cattle, WQ = WQ yak, SZ = SZ yak, ZB = ZB yak. The representative genus were based on a indicator value >0.5.

As the host significantly affected the microbiota and the effect of each stomach region was not significant, we compared the microbiota of each stomach chamber across the five breeds. A CCA was applied to characterize the representative bacteria for each stomach chamber. A total of 18, 18, 17, and 18 taxa were identified in the rumen (Figure 4A), reticulum (Figure 4B), omasum (Figure 4C), and abomasum (Figure 4D) of the five breeds, respectively. Overall, the comparison of these identified taxa showed that the four stomach chambers of dairy cattle, yellow cattle and WQ yak had a significantly higher abundance of *Prevotella* and *Succiniclasticum* spp. than those of SZ yak and ZB yak. Conversely, the four stomach chambers of SZ yak and ZB yak were characterized by high levels of *Christensenellaceae* R7 and *Lachnospiraceae* UCG 008 (Tables 2–5).

**TABLE 2:** Comparison of the relative abundance (%) of the representative bacteria^N^ at the genus level in the rumen of the five breeds.

**Rumen**	**Dairy cattle**	**Yellow cattle**	**WQ yak**	**SZ yak**	**ZB yak**	**SEM**	***P***
*Prevotella*	26.79^b^	18.00^*ab*^	23.47^b^	3.29^a^	5.62^a^	2.87	0.003
*Rikenellaceae* RC9	10.15	19.29	13.51	20.59	21.46	1.69	0.128
*Prevotellaceae* UCG 001	2.50	1.89	5.83	3.30	6.51	0.97	0.523
*Fibrobacter*	2.19	0.56	1.10	0.03	1.18	0.33	0.372
*Succiniclasticum*	2.75^*ab*^	1.89^*ab*^	4.21^b^	0.59^a^	0.77^a^	0.45	0.030
*Ruminococcus* 1	2.11	0.84	1.22	1.01	2.95	0.37	0.354
*Prevotellaceae* UCG 003	1.37	1.69	1.88	0.23	2.34	0.42	0.656
*Ruminococcaceae* NK4A214	2.71^*ab*^	2.34^a^	1.69^a^	4.98^b^	2.76^*ab*^	0.36	0.012
*Saccharofermentans*	1.86	0.75	0.61	1.89	1.02	0.19	0.078
*Ruminococcaceae* UCG 005	1.46^a^	1.46^a^	1.14^a^	3.11^b^	1.72^b^	0.25	0.047
*Lachnospiraceae* AC2044	1.04	0.58	1.42	0.47	0.51	0.18	0.411
*Christensenellaceae* R7	1.98^a^	2.09^a^	1.53^a^	15.07^b^	10.00^b^	1.55	0.001
*Ruminococcaceae* UCG 010	1.25	2.09	1.29	2.14	1.60	0.14	0.081
*Papillibacter*	0.79	1.54	0.73	1.08	1.05	0.10	0.066
*Eubacterium coprostanoligenes*	1.23^a^	1.78^*ab*^	0.54^a^	2.94^b^	1.45^*ab*^	0.25	0.007
*Lachnospiraceae* UCG 008	0.21^a^	0.29^a^	0.34^a^	1.68^b^	1.82^b^	0.23	0.014
*Butyrivibrio* 2	0.89	1.51	2.07	0.84	0.97	0.24	0.057
*Alloprevotella*	0.02^a^	0.04^a^	0.05^a^	0.90^b^	3.55^c^	0.05	0.004

*Means within the same row with different letters are significantly different from one another. N, the method of selecting the representative bacteria at the genus level is described in Section “Bioinformatics and Statistical Analyses.”*

**TABLE 3:** Comparison of the relative abundance (%) of the representative bacteria^N^ at the genus level in the reticulum of the five breeds.

**Reticulum**	**Dairy cattle**	**Yellow cattle**	**WQ yak**	**SZ yak**	**ZB yak**	**SEM**	***P***
*Alloprevotella*	0.06	0.06	0.09	1.19	2.29	0.32	0.080
*Butyrivibrio* 2	0.78^a^	2.53^b^	1.79^*ab*^	1.67^*ab*^	0.92^a^	0.20	0.017
*Christensenellaceae* R7	1.86^a^	2.76^a^	2.19^a^	16.62^b^	11.28^b^	1.67	0.000
*Fibrobacter*	3.20	0.52	1.52	0.01	0.71	0.39	0.061
*Lachnospiraceae* AC2044	0.91	0.83	1.05	0.68	0.47	0.08	0.302
*Lachnospiraceae* FCS020	0.26^a^	0.19^a^	0.35^a^	1.04^b^	2.27^b^	0.28	0.044
*Lachnospiraceae* UCG 008	0.18^a^	0.51^*ab*^	0.47^*ab*^	2.17^c^	1.67^*bc*^	0.23	0.002
*Prevotella*	31.13^b^	21.45^*ab*^	18.56^*ab*^	2.65^a^	8.16^*ab*^	3.31	0.021
*Prevotellaceae* UCG 001	3.29	2.00	5.12	2.82	7.00	1.05	0.642
*Prevotellaceae* UCG 003	1.59	1.19	1.61	0.21	2.47	0.44	0.662
*Rikenellaceae* RC9	11.78	18.23	13.89	11.53	16.99	1.76	0.731
*Romboutsia*	0.17^a^	0.20^a^	0.01^a^	1.84^b^	1.73^b^	0.28	0.028
*Ruminococcaceae* NK4A214	2.24^a^	1.97^a^	2.23^a^	5.38^b^	3.23^*ab*^	0.40	0.014
*Ruminococcaceae* UCG 005	0.88^a^	0.62^a^	1.69^a^	2.88^b^	1.45^a^	0.21	0.001
*Ruminococcus* 1	2.13	1.45	1.69	1.10	2.96	0.36	0.604
*Saccharofermentans*	1.17^a^	0.70^a^	0.79^a^	2.45^b^	0.85^a^	0.19	0.003
*Succiniclasticum*	5.28^b^	1.57^*ab*^	5.64^b^	0.61^a^	0.77^a^	0.90^a^	0.042
*Papillibacter*	0.74	1.51	0.88	1.25	0.80	0.09	0.54

*Means within the same row with different letters are significantly different from one another. N, the method of selecting the representative bacteria at the genus level is described in Section “Bioinformatics and Statistical Analyses.”*

**TABLE 4:** Comparison of the relative abundance (%) of the representative bacteria^N^ at the genus level in the omasum of the five breeds.

**Omasum**	**Dairy cattle**	**Yellow cattle**	**WQ yak**	**SZ yak**	**ZB yak**	**SEM**	***P***
*Aeriscardovia*	0.00	0.07	4.81	0.47	0.15	0.95	0.485
*Christensenellaceae* R7	2.30^a^	3.47^a^	3.09^a^	13.45^b^	14.96^b^	1.64	0.001
*Fibrobacter*	1.77	0.29	0.99	0.03	2.46	0.49	0.557
*Lachnospiraceae* UCG 008	0.29^a^	0.50^*ab*^	0.48^*ab*^	1.91^b^	1.92^b^	0.23	0.008
*Papillibacter*	0.59	1.34	0.81	1.18	1.34	0.12	0.162
*Prevotella*	19.64^b^	10.61^*ab*^	21.23^b^	2.83^a^	2.48^a^	2.39	0.002
*Prevotellaceae* NK3B31	2.55	0.73	0.91	2.10	1.02	0.28	0.138
*Prevotellaceae* UCG 001	3.72	2.38	4.47	6.54	8.10	1.11	0.559
*Prevotellaceae* UCG 003	1.32	1.50	1.77	0.21	0.94	0.21	0.172
*Rikenellaceae* RC9	19.73	16.26	11.33	14.82	17.17	1.26	0.336
*Ruminococcaceae* NK4A214	1.64^a^	2.26^*ab*^	2.59^*ab*^	5.25^b^	4.57^*ab*^	0.46	0.026
*Ruminococcaceae* UCG 005	1.53^*ab*^	1.56^*ab*^	1.10^a^	3.23^b^	2.24^*ab*^	0.25	0.042
*Ruminococcaceae* UCG 010	0.95	1.95	1.45	2.37	1.80	0.20	0.248
*Ruminococcus* 1	1.83	1.82	1.34	0.89	1.43	0.14	0.212
*Saccharofermentans*	0.78	1.17	0.96	0.93	0.72	0.06	0.221
*Succiniclasticum*	4.50^b^	1.53^b^	5.10^b^	1.08^a^	0.86^a^	0.81	0.317
*Eubacterium coprostanoligenes*	0.52^a^	2.33^*ab*^	0.95^a^	3.71^b^	2.20^*ab*^	0.35	0.007

*Means within the same row with different letters are significantly different from one another. N, the method of selecting the representative bacteria at the genus level is described in Section “Bioinformatics and Statistical Analyses.”*

**TABLE 5:** Comparison of the relative abundance (%) of the representative bacteria^N^ at the genus level in the abomasum of the five breeds.

**Abomasum**	**Dairy cattle**	**Yellow cattle**	**WQ yak**	**SZ yak**	**ZB yak**	**SEM**	***P***
*Acetobacter*	0.24	0.02	0.10	1.79	2.68	0.52	0.429
*Butyrivibrio* 2	1.56^*cd*^	1.18^*bc*^	1.89^d^	0.47^a^	0.55^*ab*^	0.15	0.000
*Christensenellaceae* R7	3.19^a^	5.79^*ab*^	3.53^a^	12.30^b^	7.29^*ab*^	1.07	0.015
*Fibrobacter*	2.26^b^	2.74^b^	2.35^b^	0.01^a^	0.65^a^	0.40	0.001
*Lachnospiraceae* UCG008	0.14^a^	0.20^a^	0.45^a^	2.08^b^	1.68^*ab*^	0.25	0.005
*Mycoplasma*	0.30^a^	0.02^a^	0.63^a^	4.49^b^	8.85^b^	1.71	0.004
*Papillibacter*	0.59	1.92	0.68	1.27	1.21	0.21	0.319
*Prevotella*	23.99^b^	6.11^*ab*^	18.38^*ab*^	1.39^a^	5.57^*ab*^	2.79	0.016
*Prevotellaceae* UCG001	2.75	1.01	4.40	3.62	6.50	0.90	0.452
*Prevotellaceae* UCG003	1.13	1.19	1.52	0.14	2.01	0.37	0.679
*Rikenellaceae* RC9	10.23	9.77	7.85	16.40	12.32	1.24	0.246
*Ruminococcaceae* NK4A214	5.18	6.10	2.89	8.54	4.04	0.75	0.142
*Ruminococcaceae* UCG005	0.81^a^	4.14^b^	1.14^a^	3.53^*ab*^	1.67^*ab*^	0.42	0.012
*Ruminococcus* 1	1.38	2.13	1.68	1.08	3.84	0.54	0.592
*Saccharofermentans*	0.57	0.88	1.12	1.07	0.60	0.10	0.365
*Succiniclasticum*	4.50^b^	1.86^b^	7.91^b^	1.25^a^	0.65^a^	1.45	0.553
*Eubacterium coprostanoligenes*	0.54^a^	3.01^*bc*^	0.94^*ab*^	3.36^c^	1.45^*abc*^	0.35	0.009

*Means within the same row with different letters are significantly different from one another. N, the method of selecting the representative bacteria at the genus level is described in Section “Bioinformatics and Statistical Analyses.”*

In the rumen (Table 2), the relative abundance of the *Ruminococcaceae* UCG 005 group was also much higher in SZ yak and ZB yak than in dairy cattle, yellow cattle and WQ yak. The relative abundance of the *Ruminococcaceae* NK4A214 group and *Eubacterium coprostanoligenes* were significantly higher in SZ yak than in the other four breeds. The relative abundance of *Fibrobacter* spp. tended to increase in the rumen of dairy cattle, and *Rikenellaceae RC9* was increased in the rumen of SZ yak and ZB yak compared with dairy cattle, yellow cattle and WQ yak. In the omasum (Table 3), R*uminococcaceae* NK4A214, *Ruminococcaceae* UCG 005 and *E. coprostanoligenes* were significantly higher in SZ yak and ZB yak than in dairy cattle, yellow cattle and WQ yak. *Ruminococcus* 1 levels tended to be higher in the omasum of dairy cattle and yellow cattle than in WQ yak, SZ yak, and ZB yak, while the *Prevotellaceae* UCG 001 group showed the opposite trend. In the reticulum (Table 4), the abundance of the *Lachnospiraceae* FCS020 group, *Romboutsia* spp. and *Ruminococcaceae* NK4A214 were significantly increased in SZ yak and ZB yak compared to dairy cattle, yellow cattle and WQ yak. SZ yak also had the highest relative abundance of *Ruminococcaceae* UCG 005 and *Saccharofermentans* spp. of all the other breeds. Dairy cattle had the highest relative abundance of *Fibrobacter* spp. of all the other breeds. In the abomasum (Table 5), SZ yak and ZB yak had higher relative abundance of *Acetobacter* spp., *Mycoplasma* spp., *Rikenellaceae* RC9, and *E. coprostanoligenes* than those of dairy cattle, yellow cattle and WQ yak; however, the latter three breeds had a significantly greater relative abundance of *Butyrivibrio* 2 and *Fibrobacter* spp. than did the former.

### Comparing the Microbiota in the Rumen, Reticulum, Omasum, and Abomasum of Three Yak Herds

**FIGURE 5:**
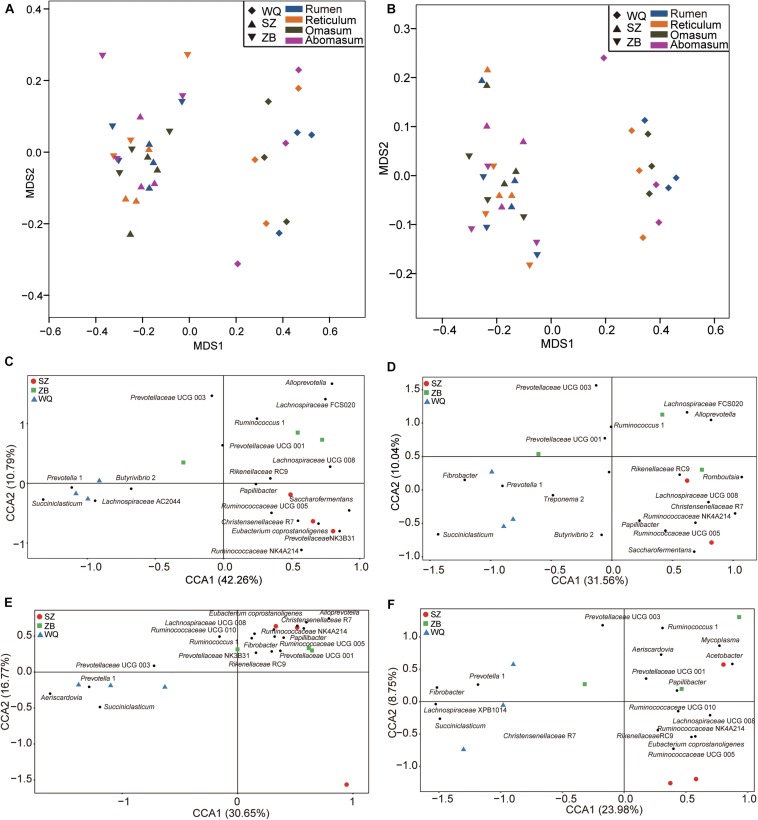
Comparing the microbial community in the four stomach compartments among three yak herds. NMDS plots of the microbiota based on the Bray–Curtis distance **(A)** and the unweighted UniFrac distance **(B)**. CCA revealing the representative bacteria at the genus level in the rumen **(C)**, reticulum **(D)**, omasum **(E)**, and abomasum **(F)** across the three yak herds. WQ = WQ yak, SZ = SZ yak, ZB = ZB yak. The representative genus were based on a indicator value >0.5.

As shown in Figure 2A, the microbiota of three yak herds also tended to separate the dairy cattle and yellow cattle, therefore, we also compared the microbiota in the four stomach chamber of three yak herds. The NMDS analysis showed that the stomach microbiota of WQ yak were significantly from that of SZ yak and ZB yak based on the Bray–Curtis distance (ANOSIM: *r* = 0.39, *p* = 0.001, Figure 5A) and the unweighted UniFrac distance (ANOSIM: *r* = 0.48, *p* = 0.01, Figure 5B). A total of 18, 18, 17, and 18 taxa were identified in the rumen (Figure 5C), reticulum (Figure 5D), omasum (Figure 5E) and abomasum (Figure 5F) of three yak herds, respectively. In the four stomach chambers, the relative abundance of *Prevotella* and *Succiniclasticum* spp. were significantly higher in WQ yak than that in SZ yak and ZB yak (Supplementary Tables S2–S5). However, *Christensenellaceae* R7 was significantly greater in SZ yak and ZB yak than that in WQ yak. *Alloprevotella* spp. showed a increasing trend in the rumen, reticulum, and omasum of SZ yak and ZB yak as compared to that in WQ yak. In addition, the relative abundance of *Lachnospiraceae* UCG 008 in the rumen and abomasum (Supplementary Tables S2, S5) and *Romboutsia* spp. in the reticulum (Supplementary Table S3), and *E. coprostanoligenes* in the omasum (Supplementary Table S4) and abomasum (Supplementary Table S5) of SZ yak and ZB yak were increased than that in WQ yak. *Acetobacter* spp., *Mycoplasma* spp., and *Rikenellaceae* RC9 were also increased in the abomasum of SZ and ZB yak as compared to that in WQ yak (Supplementary Table S5).

## Discussion

In the present study, we examined the microbiota in four stomach compartments of dairy cattle, yellow cattle and three yak herds lived in the QTP. The results demonstrated there were core bacterial composition in the fore-stomach across the five ruminant breeds. However, the findings also revealed that host genetics and geography affected the bacterial diversity and community composition in four stomach chambers, which were characterized by different taxonomy.

The present study revealed that the phyla *Bacteroidetes* and *Firmicutes* were the predominant bacteria in dairy cattle, yellow cattle, and yaks regardless of stomach region (Figure 1A). Similar to previous findings, the two phyla were also observed to be abundantly presented in the GIT of goat (Lei et al., 2018), bovine (Jami et al., 2013; Mao et al., 2015), sheep (Zeng et al., 2017), yak (Zhang et al., 2016; Zhou et al., 2017; Xue et al., 2018; Hu et al., 2019), steer (de Oliveira et al., 2013), roe deer (Li et al., 2014), indicating the ecological and functional importance of *Bacteroidetes* and *Firmicutes* in ruminant GIT. However, we found the relative abundance of *Proteobacteria* tended to increase in abomasum (2.8%) as compared to that in rumen (1.2%), reticulum (1.4%), and omasum (2.0%), which was agreement with the findings that the abomasum differed from that the other three stomach compartments in yak fed a rapid fattening regime (Xue et al., 2018). The phylum *Proteobacteria* has been identified in the rumen tissue of pre weaning calves, suggesting a role in scavenging the oxygen that facilitates microbiota colonization (Malmuthuge et al., 2014). Thus, the prevalence of phylum *Proteobacteria* in abomasum may be related to the difference of physiology and function, such as the oxygen concentration, among the four stomach chambers, resulting in the spatial heterogeneity of gut microbiota distribution (Zhang et al., 2014).

This study also showed that the genera *Prevotella* and *Rikenellaceae* RC9 were the abundant bacteria in rumen, reticulum, omasum, and abomasum of dairy cattle, yellow cattle, and three yak herds (Figure 1B), which is consistent with the previous findings in goat (Lei et al., 2018), dairy cattle (Mao et al., 2015), yak (Zhang et al., 2016; Xue et al., 2018; Hu et al., 2019), and the global rumen microbiota (Henderson et al., 2015). *Prevotella* spp. is a group of bacteria usually identified in rumen, representing the greater genetic and metabolic diversity (Bekele et al., 2010; Purushe et al., 2010), and playing major roles in carbohydrate metabolism, such as hemicellulose, starch, xylan, lignan, pectin (Dehority, 1966; Cotta, 1992; Gardner et al., 1995; Kabel et al., 2011), and nitrogen metabolism (Stevenson and Weimer, 2007; Kim et al., 2017). Although we do not know the clear function of *Rikenellaceae* RC9, however, a previous study demonstrated that *Rikenellaceae* RC9 was closely related to members of *Alistipes* spp. belonging to the family *Rikenellaceae* (Seshadri et al., 2018), which play possible roles in degrading plant derived polysaccharides (He et al., 2015; Peng et al., 2015). Taken together, these finding suggested that *Prevotella* and *Rikenellaceae* RC9 played important roles in utilizing the carbohydrate and nitrogen in the foregut of ruminants in QTP.

This study showed that the diversity index was significantly differed across the five ruminant breeds (Table 1), although the sequencing in our study did not have a higher coverage. The NMDS results revealed that the microbiota membership and structure were significantly distinct among different breeds (Figures 2A–C) whereas the obvious difference were not observed among four stomach compartments (Figure 2D). Consisting with our findings, Huang et al. (2012) found that yaks have a unique rumen microbial ecosystem that is significantly different from that of dairy cattle, and Zhang et al. (2016) also showed that the rumen microbiota of QTP ruminants significantly different from sheep and dairy cattle. These results indicated that the host genetics and geography affect the four stomach chamber microbial community. Interestingly, we found the *Firmicutes*:*Bacteroidetes* (FB) ratio were much higher in rumen (Dairy cattle = 0.33, yellow cattle = 0.30, WQ yak = 0.44, SZ yak = 1.39, ZB yak = 0.81), reticulum (Dairy cattle = 0.47, yellow cattle = 0.52, WQ yak = 0.59, SZ yak = 1.97, ZB yak = 0.92), and omasum (Dairy cattle = 0.37, yellow cattle = 0.66, WQ yak = 0.57, SZ yak = 1.30, ZB yak = 1.10) of SZ yak and ZB yak compared to that in dairy cattle, yellow cattle, and WQ yak. In previous study, the FB ratio in gut was reported to relate with human obesity (Ley et al., 2006), indicating their role in energy metabolism. A higher FB ration was also found in feces of forest musk deer fed dry leaves during spring and winter than that in summer and autumn fed fresh plants (Hu et al., 2017). Moreover, the FB ratio is increased in rumen of dairy cattle fed hay than that fed grain (Fernando et al., 2010). These results suggested that the SZ yak and ZB yak have evolved a higher capacity to utilize the fiber plants thereby supplying more energy to host at the QTP, which is further supported by the rumen metageome analysis, showing the significant enrichment in volatile fatty acids producing pathways of rumen microbial genes in high-altitude ruminants (Zhang et al., 2016).

The difference of bacterial composition at genus level was also much more evident among the five breeds (Figure 3 and Tables 2–5). *Prevotella* and *Succiniclasticum* spp. were more prevalent in dairy cattle, yellow cattle, WQ yak, whereas *Christensenellaceae* R7, *Lachnospiraceae* UCG 008, *Ruminococcaceae* UCG 005, *Ruminococcaceae* NK4A214, and *E. coprostanoligene* were abundant in SZ yak and ZB yak. To know about the metabolic function of *Prevotella*, we aligned the representative sequences of OTUs of *Prevotella* to the NCBI database, and found *Prevotella* was similar to *Prevotella copri* (92% similarity). *P. copri* is reported to contain lots of enzymes and genes involved in the fermentation and utilization of complex polysaccharides (Dodd et al., 2011), and was associated the metabolism of glucose (Kovatcheva-Datchary et al., 2015). In addition, *Prevotella* spp. was increased in the sheep rumen when fed alfalfa hay as compared to fed corn stover, which may be caused by the low content of neutral detergent soluble and crude protein (Xie et al., 2018). On the other hand, *Succiniclasticum* spp. could convert succinate to propionate which is commonly detected in the rumens of pasture fed yellow cattle (van Gylswyk, 1995). Therefore, the decreased distribution of *Prevotella* spp. and *Succiniclasticum* spp. in SZ yak and ZB yak was likely to result from the forage quality.

The bacteria within the *Christensenellaceae* family could secrete α-arabinosidase, β-galactosidase, and β-glucosidase, which were also be associated with feed efficiency (Perea et al., 2017). The bacteria belonging to the *Ruminococcaceae* family played important roles in fermenting plant fibers in the GIT (Flint et al., 2008; Biddle et al., 2013), which is documented by metagenomic (Kim et al., 2011) and transcriptomic analyses (Christopherson et al., 2014), and is associated with feed efficiency in dairy cattle (Myer et al., 2015) and lamb (Perea et al., 2017). Therefore, it is speculated that there bacteria are important for SZ yak and ZB yak adapting the harsh environment in the QTP. However, the dietary composition is also another important factor to affect the GIT microbiota (Hu et al., 2019), therefore, comparing the microbiota in the four stomach chambers of yak fed the same diet in different altitude can further improve the understanding of the role of microbiota in host adaptation.

In the present study, we found the relative abundance of *Prevotellaceae* UCG 001 tended to increase in foregut of yaks, while the proportion of *Fibrobacteria* spp. was high in dairy cattle and yellow cattle. We found the OTU sequences of *Prevotellaceae* UCG 001 were similar to *Intestinimonas butyriciproducens* (94–95% similarity). *I. butyriciproducens* could produce butyrate via the acetyl-CoA pathway and the glutamate, succinate, and lysine pathways, in which amino acids such lysine and glutamate act as substrates (Kläring et al., 2013; Bui et al., 2015). Butyrate serves as a major source of metabolic energy in ruminants and as a host signal (Bergman, 1990; Hamer et al., 2008). *Fibrobacteria* spp. is an important group of bacteria that play a key role in the degradation of cellulosic plant biomass in the rumen to produce volatile fatty acids (Tajima et al., 2001; Béra-Maillet et al., 2004; Abdul Rahman et al., 2016). Therefore, it is hypothesized that the yak may have evolved the different microbial mechanism to generate energy as compared to the dairy cattle and yellow cattle.

Importantly, the relative abundance of *Prevotella* and *Succiniclasticum* spp. were decreased with the altitude, whereas the relative abundance of *Christensenellaceae* R7, *Alloprevotella* spp., and *Lachnospiraceae* UCG 008 were increased with the altitude (Supplementary Tables S2–S5). Similarly, Das et al. (2018) also found the relative abundance of *Lachnospiraceae* was also higher in the Indian in rural high altitude. Li and Zhao (2015) also revealed that the relative abundance of *Prevotella* spp. was decreased in the gut of Chinese Han living in Tibet. These results suggested the possible role of *Prevotella* spp. in the high altitude adaptation. However, previous studies showed that the relative abundance of *Prevotella* spp. were increased in the rumen of yak (3,000–4,500 m) and Tibet sheep as compared to that of cattle and sheep (Zhang et al., 2016), and in the gut of Plateau pika (4,431 m) (Li et al., 2017). These difference may be related to the altitude of yaks in our study, which is higher (>4,500 m) than previous studies. *Alloprevotella* spp. produces moderate amounts of acetate and major amounts of succinate (Downes et al., 2013), and is reported to be associated with decreased lifetime cardiovascular disease risk (Kelly Tanika et al., 2016). Moreover, the genome analysis of *Romboutsia* spp. showed that this genus contains a versatile array of metabolic capabilities related to carbohydrate utilization and fermentation of single amino acids (Gerritsen, 2015). Moreover, *E. coprostanoligenes* is documented to have the capability of removing the cholesterol (Freier et al., 1994), and *Acetobacter* spp. could accelerate host development, increase growth rate, and help regulate host glucose and lipid levels through manipulation of host signaling pathways (Sannino et al., 2018). Therefore, these increased bacteria may help yak to adapt the high altitude through the enhanced metabolism.

In summary, our findings the genera *Prevotella* and *Rikenellaceae* RC9 were universally presented in four stomach compartments of five breeds lived in QTP, indicating the important role in foregut metabolism. However, the SZ yak and ZB yak had the significantly different microbiota as compared to that of dairy cattle, yellow cattle and WQ yak, whereas the difference among the four stomach region is limited, suggesting that the geography location and host genetics greatly affected the distribution of foregut microbiota. Moreover, the difference among these breeds were characterized by the distinctly bacterial taxonomy, indicating the potentially different mechanisms of ruminant in adaptation of QTP. However, a limitation of the present study is that the little numbers of animals is used. In later studies, more animals and metatranscriptomic analyses would further improve our understanding the microbiota role in ruminant lived on the QTP.

## Accession Numbers

The sequences from the present study have been deposited in the SRA database under the accession number SRP179146.

## Ethics Statement

All animal-specific procedures were approved and authorized by the Tibet Academy of Agricultural and Animal Husbandry Sciences Animal Care and Use Committee.

## Author Contributions

JX, ZC, CZ, HC, YZ, and QZ collected the samples. JX, ZC, and CZ analyzed the data. JZ and QJ designed the study, wrote and reviewed the manuscript. All authors approved the final version of the manuscript.

## Conflict of Interest Statement

The authors declare that the research was conducted in the absence of any commercial or financial relationships that could be construed as a potential conflict of interest.

## Supplementary Material

FIGURE S1 Statistical group distance among different foregut regions.

TABLE S1 The detailed animal information used in the present study.

TABLE S2 Comparison of the relative abundance (%) of the representative bacteria in Figure 5C at the genus level in the rumen of three yak herds.

TABLE S3 Comparison of the relative abundance (%) of the representative bacteria in Figure 5D at the genus level in the reticulum of three yak herds.

TABLE S4 Comparison of the relative abundance (%) of the representative bacteria in Figure 5E at the genus level in the omasum of three yak herds.

TABLE S5 Comparison of the relative abundance (%) of the representative bacteria in Figure 5F at the genus level in the abomasum of three yak herds.
