# Supplementary figures and images for: Comparing the Microbial Community in Four Stomach of Dairy Cattle, Yellow Cattle and Three Yak Herds in Qinghai-Tibetan Plateau
Source: Front Microbiol. 2019 Jul 10;10:1547. doi: 10.3389/fmicb.2019.01547 (PMC6636666; doi:10.3389/fmicb.2019.01547)

# Group Distances

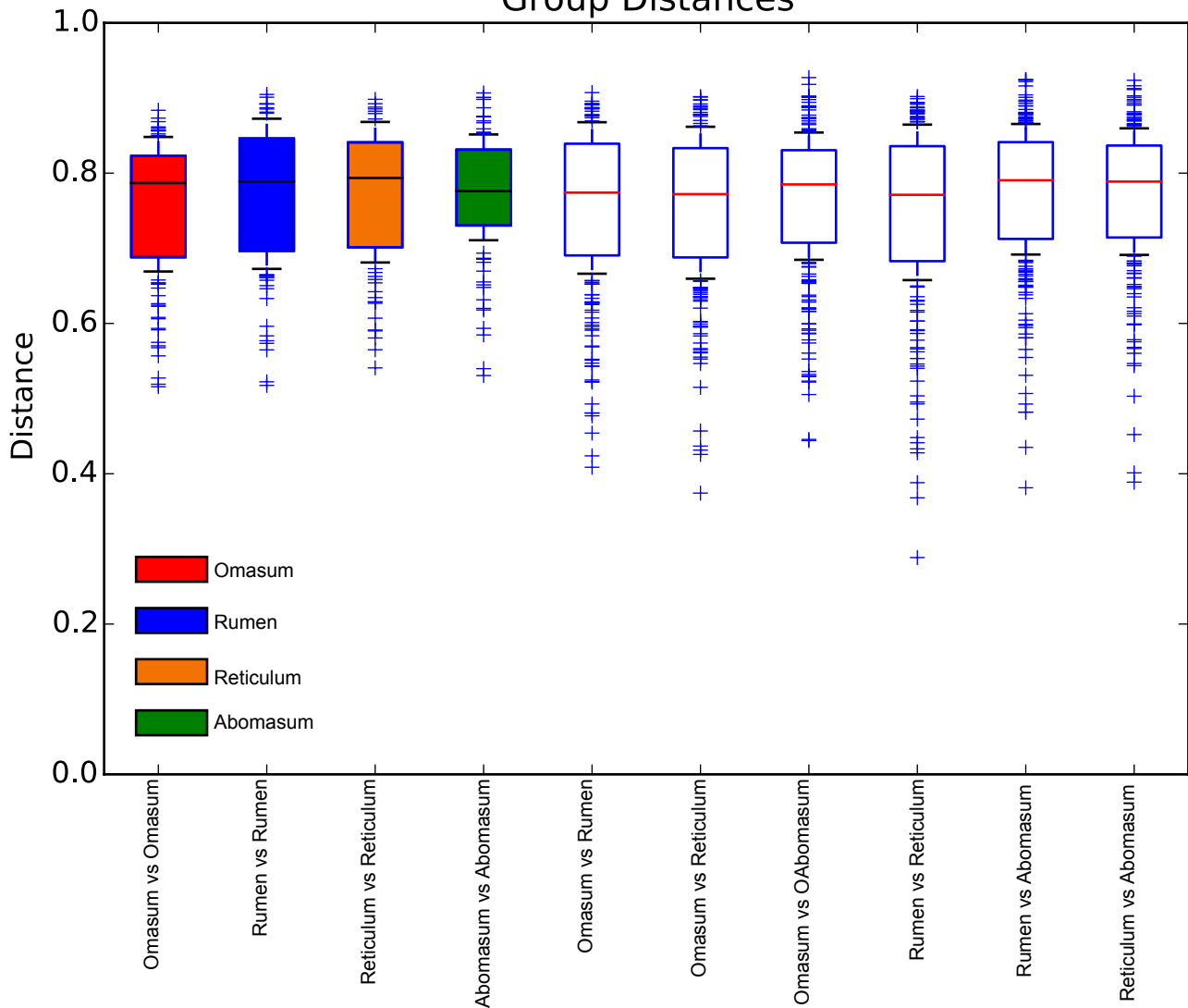

Supplement: FIGURE S1 — Statistical group distance among different foregut regions. [file Data_Sheet_1.PDF]
