# Supplementary material for: Comparing the Microbial Community in Four Stomach of Dairy Cattle, Yellow Cattle and Three Yak Herds in Qinghai-Tibetan Plateau
Source: Front Microbiol. 2019 Jul 10;10:1547. doi: 10.3389/fmicb.2019.01547 (PMC6636666; doi:10.3389/fmicb.2019.01547)
Supplement: TABLE S1 — The detailed animal information used in the present study. [file Table_1.DOCX]

**Table S1**. The detailed animal information used in the present study.

| Animal | Sex | Location | Latitude | Mean body weight | Diet |
| --- | --- | --- | --- | --- | --- |
| Holstein cattle | Female | Taiping Village, Lichun Town, Pengzhou City, Sichuan Province, China | 616 m | 674.0±25.2 kg | Concentrate and forage (Corn stover and Wheat ryegrass). The ration of concentrate and forage is 40:60 based on the dry matter. |
| Sanjiang cattle | Female | Maliu Village, Sanjiang Town, Wenchuan County, Chengdou City, Sichuan Province, China | 1,484 m | 197.7±15.6 kg | Grazing:  *Elymus dahuricus*  *Poa annua*  *Scirpus wallichii* |
| Leiwuqi (WQ) yak | Female | Riwoqê County, Qamdo City, Tibet Autonomous Region, China | 4,500 m | 210.4±33.5 kg | Grazing: Temperate meadow  *Elymus nutans*  *Kobresia humilis*  *Kobresia pygmaea*  *Carex moorcroftii* |
| Shenza (SZ) yak | Female | Shenza County, Nagchu City, Tibet Autonomous Region, China | 4,700 m | 156.0±26.7 kg | Grazing: Alpine meadow  *Kobresia pygmaea*  *Carex moorcroftii*  *Kobresia humilis*  *Potentilla fruticosa*  *Eragrostis nigra*  *Orinus anomala* |
| Zhongba (ZB) yak | Female | Zhongba County, Shigatse City, Tibet Autonomous Region, China | 4,700 m | 200.8±17.1 kg | Grazing:  *Kobresia pygmaea*  *Kobresia humilis*  *Potentilla fruticosa*  *Stipa capillacea* |
