# Supplementary material for: Comparing the Microbial Community in Four Stomach of Dairy Cattle, Yellow Cattle and Three Yak Herds in Qinghai-Tibetan Plateau
Source: Front Microbiol. 2019 Jul 10;10:1547. doi: 10.3389/fmicb.2019.01547 (PMC6636666; doi:10.3389/fmicb.2019.01547)
Supplement: TABLE S2 — Comparison of the relative abundance (%) of the representative bacteria in Figure 5C at the genus level in the rumen of three yak herds. [file Table_2.DOCX]

**Table S2**. Comparison of the relative abundance (%) of the representative bacteria at the genus level in the rumen of three yak herds.

| Rumen | WQ yak | SZ yak | ZB yak | SEM | *P* |
| --- | --- | --- | --- | --- | --- |
| *Prevotella* 1 | 23.47^a^ | 3.29^b^ | 5.62^b^ | 0.13 | 0.007 |
| *Rikenellaceae* RC9 | 13.51 | 20.59 | 21.46 | 0.16 | 0.317 |
| *Prevotellaceae* UCG 001 | 5.83 | 3.30 | 6.51 | 0.12 | 0.706 |
| *Succiniclasticum* | 4.2^a^ | 0.59^b^ | 0.77^b^ | 0.03 | 0.024 |
| *Ruminococcus* 1 | 1.22 | 1.01 | 2.95 | 0.04 | 0.351 |
| *Prevotellaceae* UCG 003 | 1.88 | 0.23 | 2.34 | 0.05 | 0.519 |
| *Ruminococcaceae* NK4A214 | 1.69^b^ | 4.98^a^ | 2.76^b^ | 0.02 | 0.008 |
| *Saccharofermentans* | 0.61^c^ | 1.89^a^ | 1.02^b^ | 0.00 | 0.001 |
| *Ruminococcaceae* UCG 005 | 1.14^b^ | 3.11^a^ | 1.72^b^ | 0.01 | 0.001 |
| *Lachnospiraceae* AC2044 | 1.42 | 0.47 | 0.51 | 0.02 | 0.349 |
| *Christensenellaceae* R7 | 1.53^b^ | 15.07^a^ | 10.00^a^ | 0.07 | 0.003 |
| *Papillibacter* | 0.73 | 1.08 | 1.05 | 0.01 | 0.475 |
| *Eubacterium coprostanoligenes* | 0.54^b^ | 2.94^a^ | 1.45^b^ | 0.01 | 0.004 |
| *Lachnospiraceae* UCG 008 | 0.34 | 1.68 | 1.82 | 0.02 | 0.105 |
| *Alloprevotella* | 0.05 | 0.90 | 3.55 | 0.06 | 0.255 |
| *Prevotellaceae* NK3B31 | 0.72 | 1.60 | 0.59 | 0.01 | 0.087 |
| *Butyrivibrio* 2 | 2.07 | 0.84 | 0.97 | 0.03 | 0.457 |
| *Lachnospiraceae* FCS020 | 0.24 | 0.98 | 2.19 | 0.03 | 0.189 |

Note. Means within the same row with different letters are significantly different from one another.
