# Supplementary material for: Comparing the Microbial Community in Four Stomach of Dairy Cattle, Yellow Cattle and Three Yak Herds in Qinghai-Tibetan Plateau
Source: Front Microbiol. 2019 Jul 10;10:1547. doi: 10.3389/fmicb.2019.01547 (PMC6636666; doi:10.3389/fmicb.2019.01547)
Supplement: TABLE S3 — Comparison of the relative abundance (%) of the representative bacteria in Figure 5D at the genus level in the reticulum of three yak herds. [file Table_3.DOCX]

**Table S3**. Comparison of the relative abundance (%) of the representative bacteria at the genus level in the reticulum of three yak herds.

| Reticulum | WQ yak | SZ yak | ZB yak | SEM | *P* |
| --- | --- | --- | --- | --- | --- |
| *Alloprevotella* | 0.09 | 1.19 | 2.29 | 0.03 | 0.201 |
| *Butyrivibrio* 2 | 1.79 | 1.67 | 0.92 | 0.01 | 0.247 |
| *Christensenellaceae* R7 | 2.19^b^ | 16.62^a^ | 11.28^a^ | 0.07 | 0.003 |
| *Fibrobacter* | 1.52 | 0.01 | 0.71 | 0.02 | 0.206 |
| *Lachnospiraceae* FCS020 | 0.35 | 1.04 | 2.27 | 0.03 | 0.204 |
| *Lachnospiraceae* UCG 008 | 0.47^b^ | 2.17^a^ | 1.67^a^ | 0.02 | 0.044 |
| *Prevotella* 1 | 18.56 | 2.65 | 8.16 | 0.20 | 0.121 |
| *Prevotellaceae* UCG 001 | 5.12 | 2.82 | 7.00 | 0.13 | 0.663 |
| *Prevotellaceae* UCG 003 | 1.61 | 0.21 | 2.47 | 0.06 | 0.516 |
| *Rikenellaceae* RC9 | 13.89 | 11.53 | 16.99 | 0.18 | 0.673 |
| *Romboutsia* | 0.01 | 1.84 | 1.73 | 0.02 | 0.104 |
| *Ruminococcaceae* NK4A214 | 2.23 | 5.38 | 3.23 | 0.03 | 0.037 |
| *Ruminococcaceae* UCG 005 | 1.69 | 2.88 | 1.45 | 0.01 | 0.027 |
| *Ruminococcus* 1 | 1.69 | 1.10 | 2.96 | 0.05 | 0.499 |
| *Saccharofermentans* | 0.79^b^ | 2.45^a^ | 0.85^b^ | 0.01 | 0.016 |
| *Succiniclasticum* | 5.64 | 0.61 | 0.77 | 0.06 | 0.068 |
| *Papillibacter* | 0.88 | 1.25 | 0.80 | 0.01 | 0.235 |
| *Treponema* 2 | 1.74 | 0.90 | 1.13 | 0.03 | 0.691 |

Note. Means within the same row with different letters are significantly different from one another.
