# Supplementary material for: Comparing the Microbial Community in Four Stomach of Dairy Cattle, Yellow Cattle and Three Yak Herds in Qinghai-Tibetan Plateau
Source: Front Microbiol. 2019 Jul 10;10:1547. doi: 10.3389/fmicb.2019.01547 (PMC6636666; doi:10.3389/fmicb.2019.01547)
Supplement: TABLE S4 — Comparison of the relative abundance (%) of the representative bacteria in Figure 5E at the genus level in the omasum of three yak herds. [file Table_4.DOCX]

**Table S4.** Comparison of the relative abundance (%) of the representative bacteria at the genus level in the omasum of three yak herds.

| Omasum | WQ yak | SZ yak | ZB yak | SEM | *P* |
| --- | --- | --- | --- | --- | --- |
| *Aeriscardovia* | 4.81 | 0.47 | 0.15 | 0.12 | 0.465 |
| *Christensenellaceae* R7 | 3.09^b^ | 13.45^a^ | 14.96^a^ | 0.10 | 0.022 |
| *Fibrobacter* | 0.99 | 0.03 | 2.46 | 0.06 | 0.505 |
| *Lachnospiraceae* UCG 008 | 0.48 | 1.91^b^ | 1.92 | 0.02 | 0.079 |
| *Papillibacter* | 0.81 | 1.18 | 1.34 | 0.01 | 0.302 |
| *Prevotella* 1 | 21.23^a^ | 2.83^b^ | 2.48^b^ | 0.11 | 0.003 |
| *Prevotellaceae* NK3B31 | 0.91 | 2.10 | 1.02 | 0.02 | 0.304 |
| *Prevotellaceae* UCG 001 | 4.47 | 6.54 | 8.10 | 0.14 | 0.739 |
| *Prevotellaceae* UCG 003 | 1.77 | 0.21 | 0.94 | 0.02 | 0.196 |
| *Rikenellaceae* RC9 | 11.33 | 14.82 | 17.17 | 0.07 | 0.132 |
| *Ruminococcaceae* NK4A214 | 2.59 | 5.25 | 4.57 | 0.04 | 0.200 |
| *Ruminococcaceae* UCG 005 | 1.10 | 3.23 | 2.24 | 0.02 | 0.077 |
| *Ruminococcaceae* UCG 010 | 1.45 | 2.37 | 1.80 | 0.02 | 0.441 |
| *Ruminococcus* 1 | 1.34 | 0.89 | 1.43 | 0.02 | 0.552 |
| *Succiniclasticum* | 5.10 | 1.08 | 0.86 | 0.07 | 0.177 |
| *Eubacterium coprostanoligenes* | 0.95^b^ | 3.71^a^ | 2.20^a^ | 0.02 | 0.036 |
| *Alloprevotella* | 0.04 | 0.47 | 1.99 | 0.03 | 0.145 |

Note. Means within the same row with different letters are significantly different from one another.
