# Supplementary material for: Comparing the Microbial Community in Four Stomach of Dairy Cattle, Yellow Cattle and Three Yak Herds in Qinghai-Tibetan Plateau
Source: Front Microbiol. 2019 Jul 10;10:1547. doi: 10.3389/fmicb.2019.01547 (PMC6636666; doi:10.3389/fmicb.2019.01547)
Supplement: TABLE S5 — Comparison of the relative abundance (%) of the representative bacteria in Figure 5F at the genus level in the abomasum of three yak herds. [file Table_5.DOCX]

**Table S5.** Comparison of the relative abundance (%) of the representative bacteria at the genus level in the abomasum of three yak herds.

| Abomasum | WQ yak | SZ yak | ZB yak | SEM | *P* |
| --- | --- | --- | --- | --- | --- |
| *Acetobacter* | 0.10 | 1.79 | 2.68 | 0.06 | 0.511 |
| *Christensenellaceae* R7 | 3.53^b^ | 12.30^a^ | 7.29^b^ | 0.09 | 0.059 |
| *Fibrobacter* | 2.35^a^ | 0.01^b^ | 0.65^b^ | 0.03 | 0.178 |
| *Lachnospiraceae* UCG 008 | 0.45^b^ | 2.08^a^ | 1.68^a^ | 0.02 | 0.083 |
| *Mycoplasma* | 0.63^b^ | 4.49^a^ | 8.85^a^ | 0.21 | 0.537 |
| *Papillibacter* | 0.68 | 1.27 | 1.21 | 0.02 | 0.506 |
| *Prevotella* 1 | 18.38^a^ | 1.39^b^ | 5.57^ab^ | 0.21 | 0.110 |
| *Prevotellaceae* UCG 001 | 4.40 | 3.62 | 6.50 | 0.11 | 0.735 |
| *Prevotellaceae* UCG 003 | 1.52 | 0.14 | 2.01 | 0.05 | 0.531 |
| *Rikenellaceae* RC9 | 7.85^b^ | 16.40^a^ | 12.32^a^ | 0.08 | 0.040 |
| *Ruminococcaceae* NK4A214 | 2.89 | 8.54 | 4.04 | 0.07 | 0.125 |
| *Ruminococcaceae* UCG 005 | 1.14^b^ | 3.53^a^ | 1.67^b^ | 0.02 | 0.017 |
| *Ruminococcus* 1 | 1.68 | 1.08 | 3.84 | 0.07 | 0.489 |
| *Succiniclasticum* | 7.91^a^ | 1.25^b^ | 0.65^b^ | 0.16 | 0.379 |
| *Eubacterium coprostanoligenes* | 0.94 | 3.36 | 1.45 | 0.03 | 0.074 |
| *Aeriscardovia* | 2.96 | 4.18 | 8.90 | 0.17 | 0.562 |
| *Lachnospiraceae* XPB1014 | 1.83 | 0.17 | 0.22 | 0.03 | 0.309 |
| *Ruminococcaceae* UCG 010 | 1.14 | 2.24 | 1.42 | 0.02 | 0.199 |

Note. Means within the same row with different letters are significantly different from one another.
